# Isocorydine Ameliorates IL-6 Expression in Bone Marrow-Derived Macrophages and Acute Lung Injury Induced by Lipopolysaccharide

**DOI:** 10.3390/ijms24054629

**Published:** 2023-02-27

**Authors:** Yifan Tu, Xiaodong Li, Yuanzheng Fu, Yunyun Chen, Hui Fang, Yuan Li, Ying Gu, Jiawei Zhang

**Affiliations:** 1 Key Laboratory of Cancer Prevention and Intervention of China National Ministry of Education, Cancer Institute, Second Affiliated Hospital, Zhejiang University School of Medicine, 88 Jiefang Road, Hangzhou 310009, China; 2 Institute of Genetics, Department of Human Genetics, Zhejiang University School of Medicine, Zhejiang University, 866 Yuhangtang Road, Hangzhou 310058, China

**Keywords:** isocorydine, lipopolysaccharide, interleukin-6, macrophages, acute lung injury

## Abstract

Isocorydine (ICD) is a type of isoquinoline alkaloid originating from *Corydalis edulis*, which has been used to relieve spasm, dilate blood vessels, and treat malaria as well as hypoxia in clinic. However, its effect on inflammation and underlying mechanisms remains unclear. The aim of our study was to determine the potential effects and mechanisms of ICD on pro-inflammatory interleukin-6 (IL-6) expression in bone marrow-derived macrophages (BMDMs) and acute lung injury mouse model. A mouse model of acute lung injury was established by intraperitoneal injection of LPS and treated with different doses of ICD. The body weight and food intake of mice were monitored to determine the toxicity of ICD. The tissue samples of lung, spleen and blood were taken to assess the pathological symptoms of acute lung injury and the expression levels of IL-6. Further, BMDMs isolated from C57BL/6 mice were cultured in vitro and treated with granulocyte-macrophage colony-stimulating factor (GM-CSF), LPS and different doses of ICD. CCK-8 assay and flow cytometry were performed to assess the viability of BMDMs. The expression of IL-6 was detected by RT-PCR and ELISA. RNA-seq was carried out to detect the differential expression genes of ICD-treated BMDMs. Western blotting was used to detect the change in MAPK and NF-κB signaling pathways. Our findings show that ICD ameliorates IL-6 expression and attenuates phosphorylation of p65 and JNK in BMDMs, and can protect mice from acute lung injury.

## 1. Introduction

Inflammation is a complex physiological response of human tissues against stimulation. The main function of inflammatory response is to eliminate pathogens, necrotic cells, senescent cells, tumor cells, and start tissue repair [1]. Although inflammation is a protective response to different injuries in the body, its over-activation will lead immune cells to attack the surrounding tissues indiscriminately, destroy normal cells, and break immune homeostasis, resulting in serious organ failure or function loss, and even leading to individual death [2]; a typical example is acute respiratory distress syndrome (ARDS). After being infected with pathogens in the lungs, immune cells, mainly alveolar macrophages, attack the alveolar tissue indiscriminately to wipe out pathogens before specific antibodies are generated [3]; its damage could lead to respiratory distress and even respiratory failure.

At present, treatments for ARDS are still limited. Steroid hormones are the drug of choice for inflammation, but long-term steroid treatment could have serious side effects, including low immunity, metabolic disorders, and induced or aggravated infection [4]. Therefore, it is urgently needed to screen new drugs targeting key players in ARDS development to reduce the inflammatory responses with minimal side effects. The alveoli are infiltrated by a large number of macrophages, over-activation of which by infection and subsequent cytokine storm are important causes of ARDS. Therefore, alveolar infiltrating macrophages are a pivotal target for the treatment of ARDS [5]. Macrophages are essential components of innate immunity as well as adaptive immunity; the main functions of macrophages include phagocytosis of pathogens and debris, antigen presentation, cytokine release and so on [6]. Production of cytokines is a major feature of altered physiological functions of macrophages. When macrophages are challenged with pathogens, they produce different inflammatory cytokines such as IL-6, TNF-α, IL-12p40 and IL-1β, which can kill pathogens directly or indirectly by activating adaptive immune system [7]. However, overexpression of these cytokines will cause the cytokine storm and lead to autoimmune diseases and, in severe cases, death [8]. For example, as a master player in the cytokine network, IL-6 promotes the differentiation of B cells into plasma cells and plays a vital role in cytokine storm [9,10,11]. Therefore, it is of great clinical significance to regulate levels of inflammatory cytokines by rational control or drug intervention.

**Figure 1:**
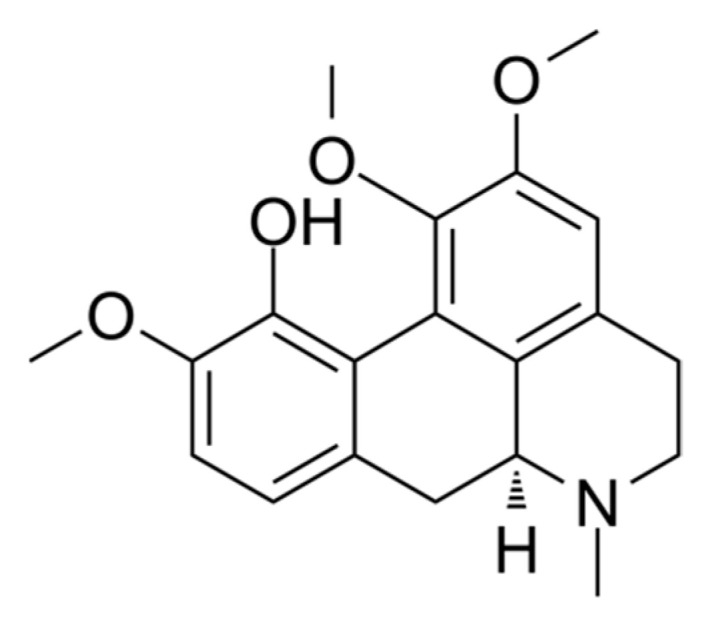
Chemical structure of Isocorydine.

Isocorydine is an isoquinoline alkaloid (Figure 1), which is extracted from the metamorphosed tuber of the poppy plant *Corydalis edulis* [12]. It is clinically used to relieve spasm, dilate blood vessels, and fight malaria, arrhythmia as well as hypoxia. In addition, its anti-tumor effects have been reported in previous studies [13,14]. There are also studies suggesting that ICD may be related to inflammation [15]. However, few investigations have been performed to study its effects on macrophage activities and LPS-induced inflammation responses. Recent studies demonstrate a key role of immune microenvironment in development of different cancers and cardiovascular diseases, which intrigues us whether ICD may regulate macrophage activities, an essential component of immune microenvironment [16].

The purpose of our study was to explores the effect of ICD on macrophage IL-6 expression stimulated by LPS, along with the efficacy and safety in vivo. With BMDMs’ activation model and LPS-induced acute lung injury mouse model, we firstly demonstrated that ICD ameliorates IL-6 expression and suppresses p65 and JNK phosphorylation in LPS-stimulated macrophages, and protects mice from acute lung injury induced by LPS.

## 2. Results

### 2.1. ICD Alleviates LPS-Induced Acute Lung Injury in Mice

**Figure 2:**
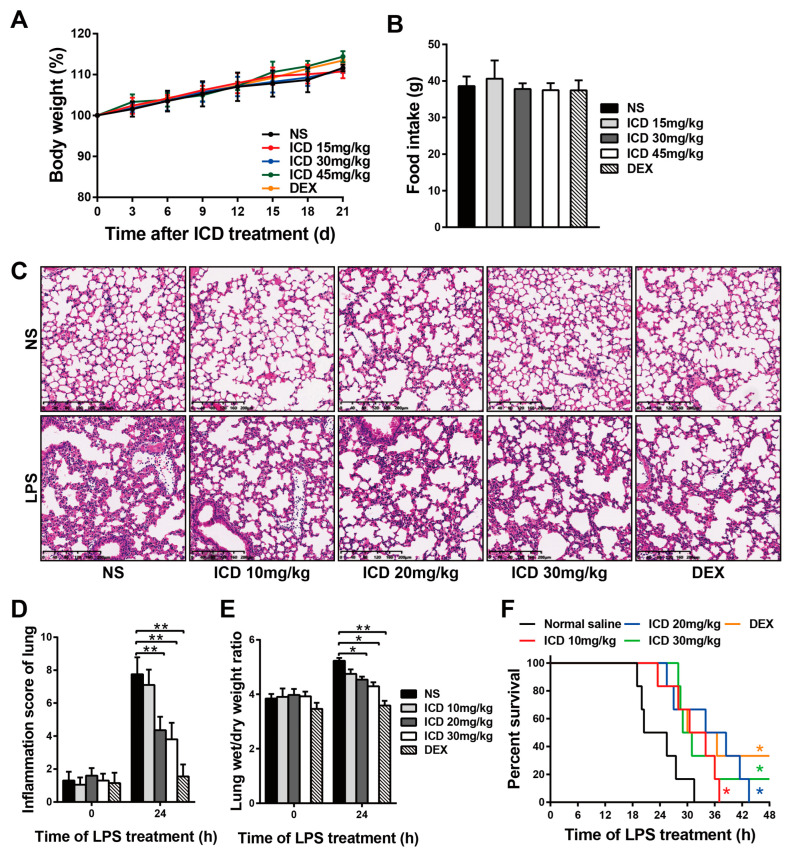
Protective effects of isocorydine on LPS-induced acute lung injury in mice. Body weight (**A**) and food intake (**B**) were monitored for 21 days in mice intraperitoneally injected with different doses of ICD and DEX (5 mg/kg). LPS-induced acute lung injury mouse model was established. (**C**) H&E staining of lung tissues. Scale bars, 200 μm. (**D**) Inflammation score of lungs. (**E**) Lung wet/dry weight ratios. (**F**) Statistics of survival time in ICD and control groups after LPS treatment. The mean and SD of at least three independent experiments are shown. * *p* < 0.05, ** *p* < 0.01 indicate significant differences between groups as determined by a two-tailed paired Student’s *t*-test.

To determine the safe dosage of ICD applied in mice, the body weight and food intake were monitored for 21 days in mice injected with different doses of ICD and dexamethasone (DEX, 5 mg/kg) for positive control. When mice were intraperitoneally injected with a low dose of ICD (<45 mg/kg), no significant weight loss and reduced food intake were observed within 21 days (Figure 2A,B). Low doses of ICD had no obvious toxic effect on the physiological state of mice.

A mouse model was established by intraperitoneal injection of 25 mg/kg LPS to determine the effect of ICD on acute lung injury. The effects of ICD on acute lung injury were detected by H&E staining. As shown in Figure 2C, LPS+normal saline (NS) group showed increased inflammatory cells infiltrating the alveoli or alveolar interstitium and thickened alveolar septum. However, the infiltration of inflammatory cells in alveolar interstitium of LPS+ICD groups was significantly less than that in LPS+NS group, and the thickness of alveolar septum was also alleviated in LPS+ICD groups. Inflammation score analysis of lung tissue showed that ICD had a significant protective effect on LPS-induced acute lung injury (Figure 2D). The degree of pulmonary edema was expressed as the ratio of lung tissue wet to dry weight. As shown in Figure 2E, the wet/dry ratios in LPS+ICD groups were lower than those in the LPS+NS group, indicating that ICD alleviated LPS-induced pulmonary edema. Furthermore, the survival time of mice in LPS+ICD groups was significantly longer than that in LPS+NS group (Figure 2F). Taken together, these data suggest that ICD alleviate LPS-induced acute lung injury.

### 2.2. ICD Inhibits IL-6 Expression in LPS-Induced Macrophage Activation and Acute Lung Injury Model

**Figure 3:**
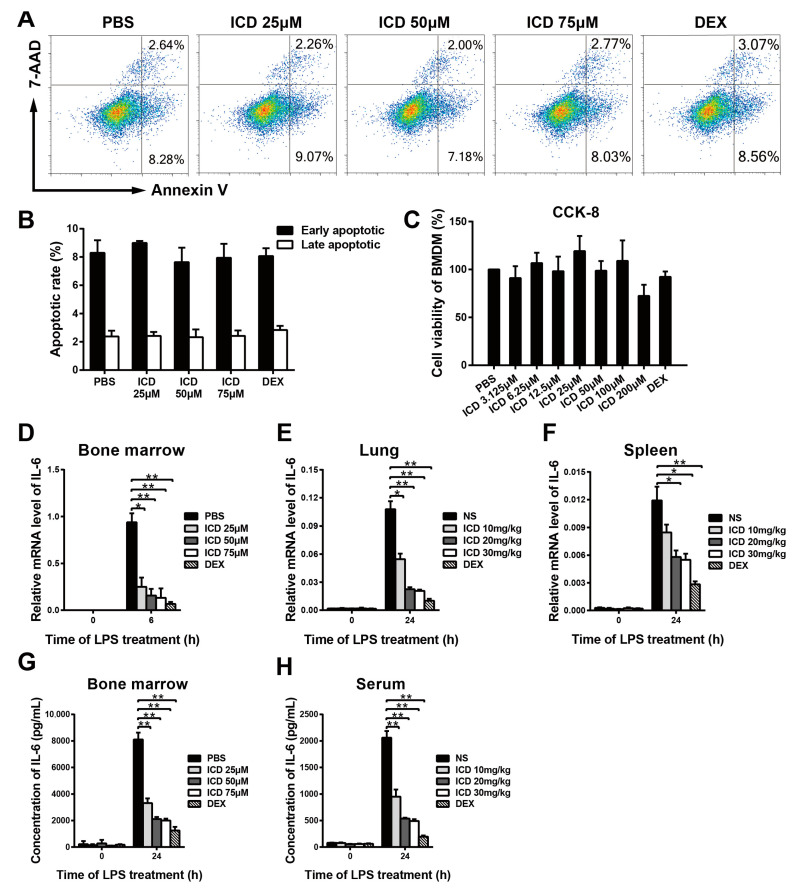
Inhibitory effects of isocorydine on IL-6 mRNA and protein expression in vitro and in vivo. Flow cytometry (**A**,**B**) and CCK-8 assay (**C**) were performed to evaluate BMDMs viability and apoptosis, after treated with different doses of ICD and DEX (5 μM) for 24 h. (**D**) The *Il6* mRNA expression in BMDMs stimulated with LPS (100 ng/mL) for 0 and 6 h. Before stimulation with LPS, BMDMs were pretreated with different doses of ICD (0, 25, 50, 75 μM) and DEX (5 μM) for 1 h. (**E**,**F**) The *Il6* mRNA expression in lung and spleen tissues of mice with LPS-induced acute lung injury. (**G**) The protein levels of IL-6 in supernatants of BMDMs stimulated with LPS (100 ng/mL) for 24 h. Before stimulation with LPS, BMDMs were pretreated with ICD (0, 25, 50, 75 μM) and DEX (5 μM) for 1 h. (**H**) The protein level of IL-6 of blood serum of mice with LPS-induced acute lung injury. In (**E**,**F**,**H**), the dose of LPS was 25 mg/kg, the doses of ICD were 0, 10, 20, 30 mg/kg and the dose of DEX was 5 mg/kg. The mean and SD of at least three independent experiments are shown. * *p* < 0.05, ** *p* < 0.01 indicate significant differences between groups, as determined by a two-tailed paired Student’s *t*-test.

To evaluate ICD toxicity to macrophages, BMDM cells were seeded into a 12-well plate and then treated with different concentrations of ICD and DEX (10 μM) for positive control for 24 h. The toxicity of ICD in BMDMs was measured by CCK-8 assay and Annexin V-APC/7-AAD apoptosis kit. The apoptotic rates are, respectively, 12.40%, 13.21%, 11.83% and 12.09% corresponding to 0, 25, 50 and 75 μM of ICD (Figure 3A,B). Similarly, no significant cytotoxicity was observed by CCK-8 assay when BMDMs were exposed to ICD with the dose less than 75 μM (Figure 3C). These results showed that the concentration of ICD below 75 μM did not affect BMDMs cell viability.

IL-6 plays a vital role in infection-induced cytokine storm and acute lung injury, which is a molecular marker of the severity of acute lung injury [17]. Therefore, we investigated whether ICD affects IL-6 production in LPS activated macrophages and acute lung injury mouse model. GM-CSF induced BMDMs were pre-treated with different doses of ICD (0, 25, 50, 75 μM) and DEX (5 μM) for positive control one hour before LPS stimulation. After stimulation with LPS for 6 h, the burst of *Il6* mRNA expression in LPS+ICD group was significantly inhibited compared to that in the LPS+PBS group, and the inhibitory effect was dose-dependent (Figure 3D). Moreover, *Il6* mRNA level was measured in lung and spleen tissue samples of acute lung injury mouse model induced by LPS with intraperitoneal injection of different doses of ICD (0, 10, 20, 30 mg/kg) and DEX (5 mg/kg) for positive control. Consistently, the *Il6* mRNA levels in LPS+ICD groups were significantly lower than those in the LPS+NS group (Figure 3E,F). These results showed that ICD suppress LPS-induced *Il6* mRNA expression in vitro and in vivo. To further confirm whether ICD affected IL-6 at the protein level, we performed ELISA assay on supernatants from LPS induced BMDMs after pretreatment with different doses of ICD and DEX (5 μM) for positive control. Compared to macrophages stimulated with LPS+PBS, macrophages treated with LPS and ICD together showed lower IL-6 protein level in 24 h (Figure 3G). Blood serum samples from acute lung injury mice were also tested with ELISA assay. Similarly, the IL-6 protein levels in LPS+ICD groups were significantly lower than those in the LPS+NS group (Figure 3H). These results showed that ICD restrain the production of IL-6 in macrophages.

### 2.3. ICD Suppresses Inflammatory Pathways in LPS-Activated Macrophages

**Figure 4:**
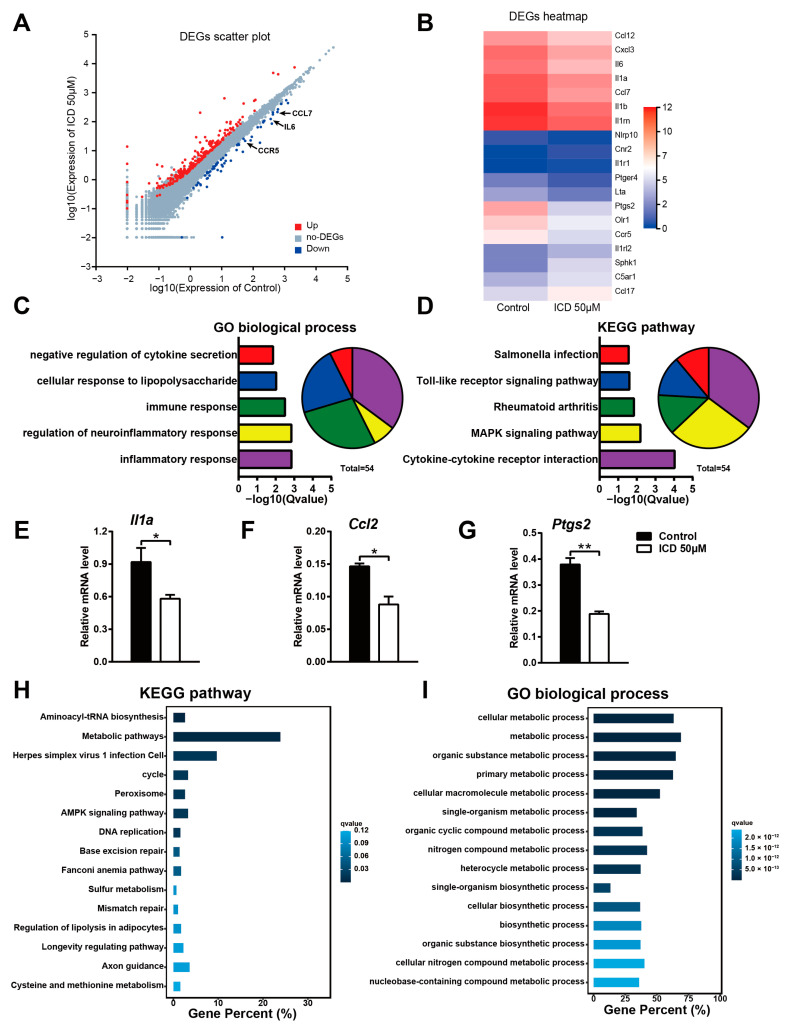
ICD compromises expression of inflammation pathway-related genes in LPS-activated macrophages. (**A**) Scatter plot showing gene expression changes in LPS triggered BMDMs pretreated ICD, compared to BMDMs treated with LPS alone. Downregulated genes are indicated in blue, while upregulated genes are indicated in red. (**B**) Heatmap of differential expressed genes involved in Inflammatory Response. The expression level is shown in the form of Log2(FPKM). (**C**) GO (Biological Process) analysis of the downregulated genes in ICD pretreated BMDMs. (**D**) KEGG analysis of the downregulated genes in ICD pretreated BMDMs. (**E**–**G**) The mRNA level of *Il1a*, *Ccl2* and *Ptgs2* in LPS stimulated macrophages for 6 h, pretreated with ICD or not. (**H**) KEGG analysis of the upregulated genes in ICD pretreated BMDMs. (**I**) GO (Biological Process) analysis of the upregulated genes in ICD pretreated BMDMs. The mean and SD of at least three independent experiments are shown. * *p* < 0.05, ** *p* < 0.01 indicate significant differences between groups as determined by a two-tailed paired Student’s *t*-test.

To uncover the mechanisms by which ICD affect inflammation activities in macrophages, we compared the transcriptomes of control and ICD-pretreated macrophages after stimulation with LPS by high-throughput sequencing of cDNA libraries (RNA-seq). Considering a |fold change| > 1.5 and FDR < 0.05 as the cutoff value, we found that 67 genes were downregulated and 263 genes were upregulated in ICD-pretreated macrophages. The downregulated genes include many pro-inflammatory genes, such as IL-6, IL1a, CCL7, CCL2, CCR5 and PTGS2 (Figure 4A,B). Then, we performed a GO analysis of the downregulated genes and found that these genes were significantly enriched in a variety of biological processes, such as Inflammatory Response, Immune Response and Cellular Response to LPS (Figure 4C). Moreover, the KEGG pathway analysis of the downregulated genes suggests a significant enrichment of inflammatory pathways, such as Toll-like receptor signaling pathway, Cytokine–cytokine receptor interaction and MAPK signaling pathway (Figure 4D). Among these DEGs, IL-1α is one of the most prominent mediators of inflammation resulting in fever and immune activation via binding to IL-1 receptor 1 [18]; CCL2 (MCP-1) is a pro-inflammatory chemokine that can mediate inflammation in multiple organs [19]; PTGS2 is an enzyme that can synthesize the pro-inflammatory mediator prostaglandins [20]. Therefore, we measured the expression of these three inflammatory genes to further confirm the RNA-seq analysis (Figure 4E–G). On the other hand, we also performed GO and KEGG enrichment analysis for upregulated genes and found that upregulated genes were mainly enriched in Aminoacyl-Trna biosynthesis, Base excision repair signaling pathways, and Fanconi anemia pathway, which were less associated with the inflammatory effects of LPS stimulation (Figure 4H,I). Therefore, these findings further support that ICD suppresses the production of key pro-inflammatory cytokines and contributes to the inhibition of inflammation.

### 2.4. ICD Reduces the MAPK and NF-κB Pathways Activated by LPS in BMDMs

**Figure 5:**
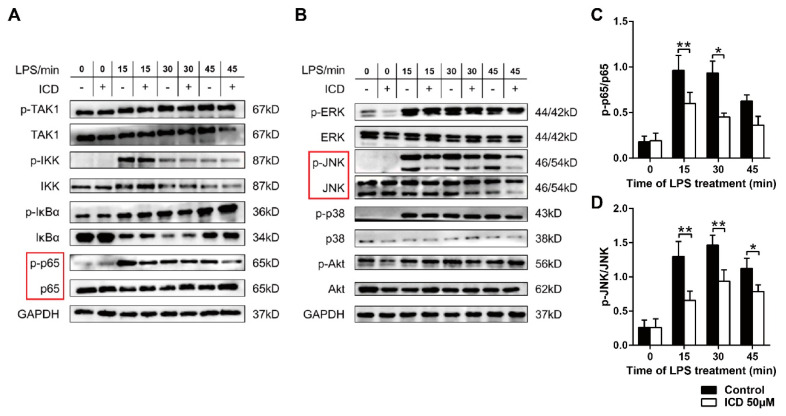
ICD reduces the activation of LPS-induced inflammation-related signaling pathway in bone marrow-derived macrophages. (**A**,**B**) Western blot analysis activation of NF-kB and MAPK signaling pathway in macrophages stimulated with LPS at different time points as indicated, pretreated with ICD or not. The ratios of p-p65/p65 (**C**) and p-JNK/JNK (**D**) were quantified with ImageJ software (v1.53). The mean and SD of at least three independent experiments are shown. * *p* < 0.05, ** *p* < 0.01 indicate significant differences between groups, as determined by a two-tailed paired Student’s *t*-test.

RNA-seq analysis showed that down-regulated genes mainly enriched in classic inflammatory pathways including Toll-like receptor signaling pathway and the biological process of Cellular Response. As a member of Toll-like receptors, Toll-like receptor 4 (TLR4) is a pattern recognition receptor on the surface of macrophages associated with bacterial immunity, and mediated LPS-induced signal transduction in most Gram-negative bacteria [21]. To study the mechanisms of the functions of ICD we investigated the effect of ICD on TLR4 signaling pathway. The results showed that ICD significantly inhibit the phosphorylation levels of p65 (Figure 5A,C), a key post-translational modification, to promote p65′s translocation from cytoplasm to nucleus and transcriptional activities [22]. Meanwhile, we also examined activation of ERK, JNK, p38 and Akt by Western blot. The results showed that ICD significantly inhibit the phosphorylation of JNK (Figure 5B,D), which is an important component of activator protein 1 (AP1) transcription factor complex [23], while the phosphorylation levels of ERK, p38, Akt, TAK, IκB and IKK were not affected. These results showed that ICD reduces the phosphorylation levels of p65 and JNK to dampen the NF-kB and MAPK pathways, and eventually affects the transcription profile of macrophages induced by LPS.

## 3. Discussion

Although some important progress has been made in the treatment of acute lung injury in recent years, the therapeutic methods for acute lung injury are still limited [24]. The clinical symptoms of acute lung injury include increased microvascular permeability, alveolar and interstitial edema, hyaline membrane formation and atelectasis. After the onset of acute lung injury, immune cells accumulate in the lung, which may exert excessive immune function and attack alveolar epithelial cells or pulmonary capillary endothelial cells indiscriminately, leading to respiratory distress in patients. Inflammatory cytokine storm is an important causes of acute respiratory distress syndrome [3]. The LPS-induced mouse model of acute lung injury can be used to study a range of potential specific and non-specific targets for ARDS drug intervention [25]. Based on this model, we found that ICD could significantly inhibit acute lung injury with ignorable side effects in vivo.

The present study investigated a novel application of ICD in treatment for inflammatory diseases. ICD is an effective component extracted from *Corydalis edulis*, which is a species of plant previously used in traditional Chinese medicine [26]. ICD has been clinically used to treat heart diseases, spasm and malaria [12]. Recent studies have also shown that ICD can inhibit the occurrence and progress of some tumors [14]. Our study demonstrated for the first time that ICD ameliorates IL-6 expression in LPS-induced bone marrow-derived macrophages activation in vitro and LPS-induced acute lung injury in mice, and there is no obvious toxicity in vivo.

Interleukin-6 (IL-6) is a dominant player in the cytokine network, which not only promotes the differentiation of immune cells, but also plays a vital role in cytokine storm, acute lung injury and tumor immunity [10]. Monoclonal antibodies against IL-6 have been applied in the treatment of rheumatoid arthritis, indicating its important role in systemic inflammation or autoimmune diseases [27]. In addition to cytokines, chemokines and prostaglandins also play vital roles in inflammation [28]. In our study, we preliminarily found that ICD can suppress the LPS-induced upregulation of many cytokines and chemokines, including IL-6, IL1a, CCL7, CCL2, CCR5 and PTGS2.

It has been reported that the expression of inflammatory cytokines can be regulated by NF-κB and MAPKs signaling pathway [29,30,31]. LPS can bind to TLR4, a pattern recognition receptor on the surface of macrophages, and then activate MAPKs/NF-κB signaling pathway [21,32]. RNA-seq analysis in our study indicated that Toll-like receptor and MAPKs signaling pathway were inhibited by treatment of ICD, along with decreased expression of inflammatory cytokines. In addition, after ICD treatment, the downregulated genes were highly enriched in the biological processes of LPS-induced inflammatory response, immune response, and cellular response. Further, inhibition of MAPK and NF-κB pathways was confirmed by reduced phosphorylation of p65 and JNK in BMDMs pre-treated with ICD.

There were no up-regulated genes significantly associated with LPS-induced inflammation according to the results of RNA-seq. We found that upregulated genes were mainly enriched in Aminoacyl-Trna biosynthesis, Cell cycle, DNA replication, Base excision repair signaling pathways and Fanconi anemia pathway, which were tightly associated with tumor proliferation, cycle and apoptosis, consistent with the previous reports [14]. The GO and KEGG pathway analysis of upregulated genes also suggest other possible biological processes such as anti-virus and cellular metabolism, which needs to be further investigated. However, our study did not identify the direct target of ICD on molecular level, and further studies are needed to investigate the protein target of ICD, which will provide the basis for high-efficacy ICD derivatives development.

**Figure 6:**
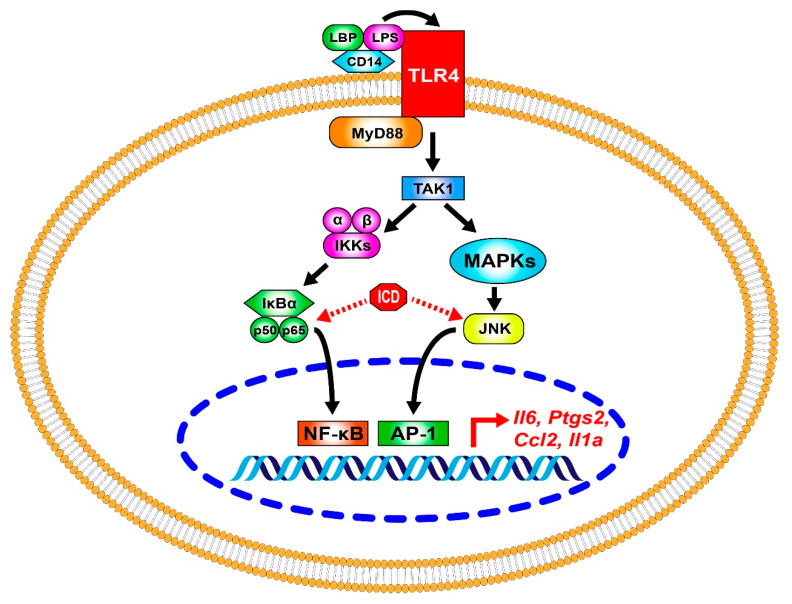
Schematic diagram of ICD effects in macrophages.

In summary, we firstly presented that ICD ameliorates IL-6 expression and regulates the phosphorylation levels of p65 and JNK in LPS-induced bone marrow-derived macrophages activation and protected mice from LPS-induced acute lung injury (Figure 6). Our study indicate that ICD might have great potential in the treatment of acute lung injury, and several studies are needed to explore other important physiological effects of ICD in future.

LPS binds to TLR4 and initiates the downstream cascade. TAK1-IKK-p65 signaling pathway activates the transcription factor NF-κB. TAK1 can also activate MAPKs, including ERK, JNK and p38, which further activate the transcription factor AP-1. ICD reduced the phosphorylation of p65 and JNK and suppressed the NF-κB and AP-1 mediated transcriptional activation of genes like IL-6, IL1a, CCL2 and PTGS2.

## 4. Materials and Methods

### 4.1. Isocorydine

Isocorydine (purity ≥ 98%) used in this study was purchased from Pufei De Biotechnology Ltd. (Chengdu, China). It has the chemical formula C_20_H_23_NO_4_ and a molecular weight of 341.406.

### 4.2. Animals

C57BL/6 mice (5 weeks old) were purchased from SLAC Laboratory Animal Ltd. (Shanghai, China). The mice were raised less than five/cage with standard chow and water under suitable temperature and humidity, the dark-lighting cycle was carried out for 12 h.

### 4.3. Cell Preparation and Culture

We prepared and cultured bone marrow-derived macrophages [33]. BMDMs were generated in RPMI-1640 medium (Gibco, Waltham, MA, USA) containing 10 ng/mL recombinant mouse GM-CSF (PeproTech, Rocky Hill, CT, USA), 10% fetal calf serum (Gibco, Waltham, MA, USA), and 100 U/mL penicillin-streptomycin (Gibco, Waltham, MA, USA) for 6 days with replacement of the culture medium every 2 days.

### 4.4. Drug Preparation

ICD and DEX (MCE, Monmouth Junction, NJ, USA) were dissolved in dimethyl sulfoxide (Sangon Biotech, Shanghai, China). Before stimulation with 100 ng/mL LPS (Sigma-Aldrich, St. Louis, MO, USA) dissolved in phosphate buffered saline (Gibco, Waltham, USA), ICD was added to the culture medium and incubated for 1 h. Before intraperitoneal injection, ICD, DEX and LPS solution were dissolved in normal saline (Sangon Biotech, Shanghai, China).

### 4.5. Cell Viability Assay

BMDMs were seeded in 96-well plates (Corning, New York, NY, USA) at 2 × 10^5^ cells/mL. The cells were treated with ICD at different doses (0–200 μM) for 24 h. Then 20 μL of CCK-8 (Beyotime, Shanghai, China) was added to each well after treatment for 24 h, and the samples were detected with a microplate reader (BioTek, New York, NY, USA) at 450 nm after incubation for 2 h at 37 °C.

### 4.6. Cell Apoptosis Assay

Annexin V-APC/7-AAD apoptosis kit (Beyotime, Shanghai, China) was used in the study. Cells obtained from macrophages were stained for 20 min at room temperature with Annexin V-APC and 7-AAD, and then analyzed with a flow cytometer (ACEA, San Diego, CA, USA, Biosciences). The flow cytometry data were analyzed with NovoExpress software (v1.5.0).

### 4.7. Establishment of Mouse Acute Lung Injury Model

We established mouse acute lung injury model by intraperitoneal injection of LPS (25 mg/kg). After being intraperitoneally injected with different doses of ICD for 1 h, body weight, food intake and survival time of mice were monitored. Blood serum, lung and spleen were taken from the mice to test IL-6 and assess acute lung injury.

### 4.8. H&E Staining of Lung Tissues

The lobes of lung were taken and fixed with 4% paraformaldehyde solution (Beyotime, Shanghai, China) for 24 h. Then, the lobes of lung were prepared into paraffin sections and stained with H&E staining kit (Solarbio, Beijing, China). The thickness of lung tissue sections was 10 μm. The pathological changes in the lung were observed under a light microscope. Lung injury scores were assessed by different researchers in blinded manner [34]. According to the Smith score system, the severity of lung injury was scored according to five indexes including pulmonary edema, alveolar and interstitial inflammatory cell infiltration, alveolar and interstitial hemorrhage, atelectasis, and hyaline membrane formation [35].

### 4.9. Lung Wet/Dry Ratios

The whole lungs of mice were taken to measure the wet weight, dried and dehydrated in an oven at 60 °C for 48 h, and then weighed again to calculate the wet/dry ratios of whole lung tissues.

### 4.10. Real-Time Quantitative Polymerase Chain Reaction

We isolated total RNA of lungs, spleens and macrophages using RNAfast200 kit (Fastagen Biotech, Shanghai, China) according to the manufacturer’s instructions. PrimeScript^TM^ RT Reagent Kit (TAKARA, Shiga, Japan) was used in cDNA synthesis. IL-6, IL1a, CCL2 and PTGS2 gene expressions were quantified by quantitative real-time PCR (LC480, Roche, Basel, Switzerland) using SYBR Green (TAKARA, Shiga, Japan), and normalized to *Gapdh* mRNA. Primers were as follows:

*Gapdh*-RTF, CTGAGTATGTCGTGGAGTCT;
*Gapdh*-RTR, GTGGATGCAGGGATGATGTT;
*Il6*-RTF, AGTTGCCTTCTTGGGACTGA;
*Il6*-RTR, TCCACGATTTCCCAGAGAAC;
*Il1a*-RTF, GTTCTGCCATTGACCATCTC;
*Il1a*-RTR, CAGAATCTTCCCGTTGCTTG;
*Ccl2*-RTF, GTGTCCCAAAGAAGCTGTAG;
*Ccl2*-RTR, CACATTCAAAGGTGCTGAAGA;
*Ptgs2*-RTF, GCGACATACTCAAGCAGGAGCA;
*Ptgs2*-RTR, AGTGGTAACCGCTCAGGTGTTG.

### 4.11. Enzyme-Linked Immunosorbent Assay

We collected cellular supernatants from BMDMs and blood serum from acute lung injury mouse after treatment with different doses of ICD, and measured the IL-6 cytokine levels in supernatants by ELISA using a cytokine-specific assay kit (Thermo Fisher, Waltham, MA, USA) following the manufacturer’s instructions.

### 4.12. RNA-Seq and Bioinformatics Analysis

Total RNA was extracted from BMDMs with TRIzol reagent (Thermo Fisher, Waltham, MA, USA). Library construction and mRNA sequencing were carried out by the BGI Technology Company (Wuhan, China). The sequencing data were filtered with SOAPnuke (v1.5.2) [36]. Reference genome alignment was executed by HISAT2 (v2.0.4) [37]. Sequencing reads were successfully aligned to the reference coding gene set by Bowtie2 (v2.2.5) [38]. RSEM (v1.2.12) was applied to calculate gene expression levels [39]. Gene filtering and summing over technical replicates were done with edgeR. Differential expression analysis was carried out by edgeR to identify DEGs. The cutoff for identifying DEGs was set as the FDR < 0.01 and |log2-fold change| ≥ 2. The most significant differentially expressed genes were scaled for heatmap visualization, with mitochondrial genes and undefined genes removed. To investigate the possible mechanisms of the effects of ICD on LPS-induced macrophage inflammation, we performed GO and KEGG enrichment analysis on DEGs using clusterProfiler [40]. The cutoff for terms from enrichment analysis was FDR < 0.05.

### 4.13. Western Blot

Laemmli sample buffer (Bio-Rad, Hercules, CA, USA) was used to extract total protein from BMDMs. BCA protein assay kit (Thermo Fisher, Waltham, MA, USA) was used to detect concentration protein. Equal amounts (30 μg) of protein were separated by 10% SDS-PAGE and transferred to NC membranes. The membranes with protein were incubated with 5% milk in tris-buffered saline (TBS) containing 0.1% Tween 20 (1 × TBST) for 1 h at room temperature. For immunodetection, NC membranes were incubated with primary antibody overnight at 4 °C. After washing with 1 × TBST buffer, the membranes were incubated with anti-mouse or anti-rabbit IgG HRP-labeled secondary antibody (HuBio, Hangzhou, China) for 1 h at room temperature. Protein bands were detected by enhanced chemiluminescence (Thermo Fisher, Waltham, MA, USA) using a chemiluminescence detection system (Azure biosystems, Dublin, OH, USA) and. The Western blot results analysis was performed using ImageJ software (v1.53).

### 4.14. Statistical Analysis

The mean and SD of at least three independent experiments were shown, except where otherwise indicated, and comparisons were performed by a two-tailed paired Student’s *t*-test. Statistical analysis was performed using GraphPad Prism (v7.0.0) software. Significance levels (*p* values) are presented in the figures, where *p* < 0.05 was considered statistically significant.

## Data Availability

The data presented in the study are available from the corresponding authors.
